# Endogenous L-Carnosine Level in Diabetes Rat Cardiac Muscle

**DOI:** 10.1155/2016/6230825

**Published:** 2016-04-13

**Authors:** Yali Liu, Dan Su, Ling Zhang, Shaofeng Wei, Kuangyi Liu, Mi Peng, Hanyun Li, Yonggui Song

**Affiliations:** ^1^Jiangxi University of Chinese Traditional Medicine, 56 Yangming Road, Nanchang 330006, China; ^2^Science and Technology College, Jiangxi University of Chinese Traditional Medicine, 819 Xingwan Road, Nanchang 330008, China

## Abstract

A novel method for quantitation of cardiac muscle carnosine levels using HPLC-UV is described. In this simple and reliable method, carnosine from the rat cardiac muscle and the internal standard, thymopentin, were extracted by protein precipitation with acetonitrile. The method was linear up to 60.96 *μ*g·mL^−1^ for L-carnosine. The calibration curve was linear in concentration ranges from 0.5 to 60.96 *μ*g·mL^−1^. The relative standard deviations obtained for intra- and interday precision were lower than 12% and the recoveries were higher than 90% for both carnosine and internal standard. We successfully applied this method to the analysis of endogenous carnosine in cardiac muscle of the diabetes rats and healthy control rats. The concentration of carnosine was significantly lower in the diabetes rats group, compared to that in the healthy control rats. These results support the usefulness of this method as a means of quantitating carnosine and illustrate the important role of L-carnosine in cardiac muscle.

## 1. Introduction

**Figure 1:**
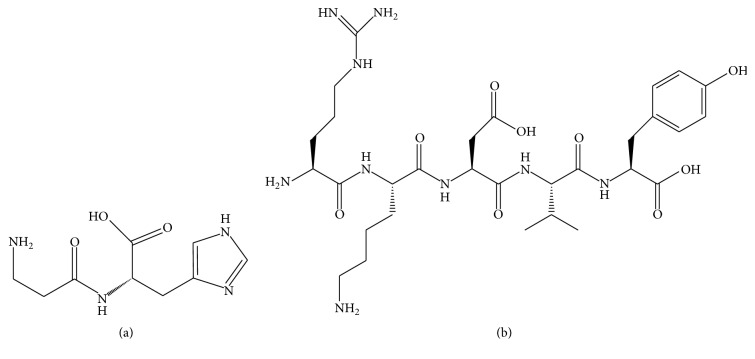
Chemical structures of L-carnosine (a) and thymopentin ((b), IS).

Carnosine (Figure 1(a)), N-beta-alanyl-L-histidine, an endogenous material, has been found in skeletal muscle [1, 2], brain, olfactory bulbs [3], and crystalline lens [4, 5]. It possesses antioxidant properties and has been shown to scavenge oxygen free radicals [6], protect mitochondrial membranes from free radical damage [7], decrease lipid peroxidation of cell membranes [8–11], and have inotropic properties [12, 13]. Studies have also revealed that carnosine can act on myocardial integrity in rats following hemorrhage induced myocardial ischemia [14] and has beneficial cardiac effects [15].

With its high content in animal tissues, carnosine is the muscle food that has been postulated to be a bioactive component. In view of the growing interest in and the biological importance of carnosine, there is a definite need for a sensitive, specific, and simple analytical method to detect it in biological matrices. The current methods for measuring carnosine in biologic matrices include micellar liquid chromatography [2], high-performance anion-exchange chromatography [16, 17], HPLC-MS [18], and HPLC-UV with precolumn derivatization [19, 20] and without derivatization using NH_2_ column [1]. All these methods suffer from several drawbacks: tedious micellar mobile phases preparation and care of the column procedure, instability of derivatives, and the high cost of MS detector preventing their utilization by many laboratories. The most recent methods (HPLC) have been assayed without internal standard affecting accuracy of the analytical method.

We developed a simple and reliable analytical method that combined HPLC with an inexpensive UV detector to quantify endogenous carnosine in rat tissues. We then successfully applied this method to the analysis of carnosine in the diabetes rat cardiac muscle.

## 2. Experimental

### 2.1. Chemicals and Reagents

L-carnosine (purity > 99.1%) (Sigma Chemicals, St. Louis, MO, USA); the crude drug of L-carnosines (Fisher Chemicals Reagent Co., Ltd., Jinan, China); thymopentin (IS) (Figure 1(b)) (Sinopharm Chemical Reagent Co., Ltd., Shenyang, China); potassium phosphate and potassium dihydrogen phosphate (Damao Chemical Reagent Factory, Tianjin, China); and acetonitrile and methanol were of HPLC grade. All the other reagents were of analytical grade. Water was triple distilled and was passed through a 0.22 *μ*m filter prior to use in all studies. Wistar rats (Experimental Animal Center of Shenyang Pharmaceutical University, Shenyang, China) were used.

### 2.2. Apparatus and Chromatographic Conditions

The HPLC system consisted of a Hitachi L-2130 pump and L-2400 UV detector. The separation was performed on Elite NH_2_ column (200 mm × 4.6 mm, 5 *μ*m) (Elite Corp., Dalian, China), protected by ODS guard column (10 mm × 4.6 mm i.d., 5 *μ*m), using acetonitrile–32 mM potassium dihydrogen phosphate, 6 mM potassium hydrogen phosphate, and 5% (*v/v*) ammonia water buffer (25 : 75,* v/v*) as mobile phase at a flow rate of 1.0 mL min^−1^. The column temperature was maintained at 35°C. The wavelength was 210 nm. A personal computer equipped with a Hitachi chromatopac D-2000 Elite program for LC systems was used to acquire and process chromatographic data. The concentration of carnosine in each sample was determined by calculating the ratio of the peak area of carnosine to that of the IS and comparing it to the calibration curve for each HPLC run.

### 2.3. Animals and Diabetic Induction

10 male Sprague Dawley (SD) rats (200 ± 10 g) were obtained from the Experimental Animal Center of Shenyang Pharmaceutical University. Animals were housed under controlled conditions (22 ± 2°C, RH 50 ± 20%) with a natural light-dark cycle for 3 days before the experiment carried out. Rats were fasted overnight and allowed free access to water. All procedures involving animals were in accordance with the Regulations of Experimental Animal Administration issued by the State Committee of Science and Technology of China. After 3 days of acclimatization, the rats were randomly divided into 2 groups (*n* = 5/group). In Diabetes Group (DG), the rats were treated with streptozotocin (40 mg/kg bodyweight in 0.1 M citrate buffer, pH 4.5) i.p. for 5 consecutive days. Blood glucose level was monitored on days 3 and 7 from the tail vein. Seven days after streptozotocin treatment, rats with fasting blood glucose levels over 13.8 mmol/L were defined as diabetic rats. In Healthy Control Group (HCG), the rats were given 0.1 M citrate buffer i.p. with approximately the same volume water as the Diabetes Group.

### 2.4. Sample Collection and Preparation

The animals were killed by decapitation and the cardiac tissues were removed and stored in liquid nitrogen until processed. The rat cardiac muscle was homogenized in cold PBS (1 : 3,* w/v*) containing protease inhibitor cocktail (50 *μ*g·g^−1^ wet tissue). The homogenates were stored frozen at −20°C until analysis.

The internal standard thymopentin 20 *μ*L and acetonitrile 400 *μ*L were added to 200 *μ*L of cardiac muscle homogenates. The mixture was vortexed for 3 min and centrifuged at 16350 g for 5 min. The 20 *μ*L of the supernatant was used for analysis.

### 2.5. Preparation of Stock Solutions, Working Solutions, and Quality Control Samples

The stock solutions of carnosine and IS were prepared in distilled water at concentration levels of 1000 *μ*g·mL^−1^, respectively. Working solutions were prepared by diluting the stock solutions with distilled water. The concentration of working solution for internal standard was 500 *μ*g·mL^−1^. The L-carnosine and IS solutions were stored at 4°C. The calibration curve of carnosine was at concentrations of 0.51, 1.02, 2.54, 10.16, 20.32, 40.64, and 60.96 *μ*g·mL^−1^ which were prepared by spiking appropriate amount of the standard solution in diluted blank cardiac muscle homogenates. Three levels of QC samples 1.02, 8.13, and 32.51 *μ*g·mL^−1^ in diluted blank cardiac muscle homogenates were prepared separately.

### 2.6. Analytical Method Validation

Carnosine and the internal standard (IS), thymopentin, were each dissolved in triple distilled water to make up a concentration of 1000 *μ*g·mL^−1^. Standard solution of carnosine was diluted at 609.6 *μ*g·mL^−1^, and the IS solution was diluted at 500 *μ*g·mL^−1^.

Blank samples were prepared by using the cardiac muscle homogenates which were diluted with triple distilled water to prevent interference by endogenous carnosine. Preliminary tests of 1 : 20 sample dilution showed the peak of endogenous carnosine under the limit of detection. The blank samples were used as matrices for calibration and QC preparation.

The calibration curve was obtained by plotting the area ratio of carnosine and IS as a function of the carnosine concentration using least squares linear regression analysis. The LOQ was defined as a reproducible concentration (*S*/*N* > 10), and the RSD% of 6 injections was below 20%.

To assess the intra- and interday accuracy and precision of the method, three concentrations levels of carnosine 1.02, 8.13, and 32.51 *μ*g·mL^−1^ for carnosine were in diluted blank cardiac muscle homogenates, with 6 replicates independently prepared at each concentration. Accuracy was defined as the relative error (RE%) while precision was defined as the relative standard deviation (RSD%).

The recovery of carnosine at three concentrations levels of 1.02, 8.13, and 32.51 *μ*g·mL^−1^ for carnosine was determined in six occasions by comparing the peak areas of carnosine from extracted samples with those in postextracted blank cardiac muscle samples spiked with carnosine at the same concentration. The recovery of IS was determined in the same way at the concentration of 25 *μ*g·mL^−1^.

The quality control (QC) samples (six replicates of QC samples at 1.02, 8.13, and 32.51 *μ*g·mL^−1^ concentrations) were assayed under room temperature (25°C) for 24 h, after three cycles (−20°C/room temperature), and long term stability for 1 month to assess the stability of carnosine in rat cardiac muscle. The resulting concentrations were compared with their theoretical concentrations, and the relative error (RE%) was calculated. Samples were concluded to be stable if the relative error was within ±15%.

### 2.7. Statistical Analysis

All values are expressed as mean ± SD. Statistical comparisons between the DG and HCG were carried out using a one-way ANOVA. A *P* value of 0.05 was considered as the threshold for a significant difference.

## 3. Results and Discussion

### 3.1. Sample Preparation

Liquid-liquid extraction and protein precipitation are the commonly used technique for sample preparation. In view of the liquid-liquid extraction usually offering much cleaner sample, various organic reagents have been tried for liquid-liquid extraction method but the great polarity of carnosine should lead to the failure. Solid-phase extraction (Strata-X cartridges (Phenomenex)) has been used for the extraction of carnosine for cleaner sample; however, the recovery in biologic matrices was too low. Therefore, protein precipitation with acetonitrile was adopted as a simple, efficient method for extracting carnosine from rat cardiac muscle and was also suitable for the extraction of IS. The recovery for both carnosine and IS is above 90%.

### 3.2. Optimization of the Chromatographic Separation

L-carnosine, containing ionizable moieties, can be too polar to be retained by the universal C_18_ column. To resolve the problem, we use NH_2_ column under reversed-phase mode with mobile phase of acetonitrile-phosphate buffer system. The adoption of phosphate buffer was found to be essential to obtain the suitable retention time. In the mobile phase without phosphate buffer, carnosine had nearly no retention and was almost eluted in dead time. The adoption of ammonia water was found to minimize the width of the carnosine peak. By increasing the amount of acetonitrile, the total elution time was decreasing but carnosine peak was closer to the other endogenous interference. The chromatographic conditions we optimized assure the appropriate resolution time of L-carnosine.

### 3.3. Choice of IS

To quantify carnosine in biological samples, several HPLC methods have also been developed. However, these measurements are hindered by lack of internal standard. As L-carnosine is a high polar analyte, without derivatization, searching for an exogenous compound whose physical and chemical properties are similar to it was an arduous and hard process. Based on our previous work, dipeptide compounds such as glycyl-L-isoleucine and L-alanyl-L-glutamine whose structures are similar to carnosine were not suitable for its chromatographic peak separation avoiding the endogenous interference. Finally thymopentin, a peptide whose polarity was less than carnosine had a suitable retention time and was sufficiently well separated from the target analyte. In an attempt to select suitable IS, a large number of dipeptides and great polar compounds have been tried, but the result was bad. Some were not retained by the NH_2_ column, and others were not separated from endogenous matrices. Thymopentin had a suitable retention time and was sufficiently well separated from the target analyte. Thymopentin was finally selected as the ideal IS for its good chromatographic behavior and stable recovery. The analytical method we adopted was sensitive, selective, reproducible, accurate, and convenient.

### 3.4. Validation of the Method

#### 3.4.1. Specificity, Linearity, and Sensitivity

**Figure 2:**
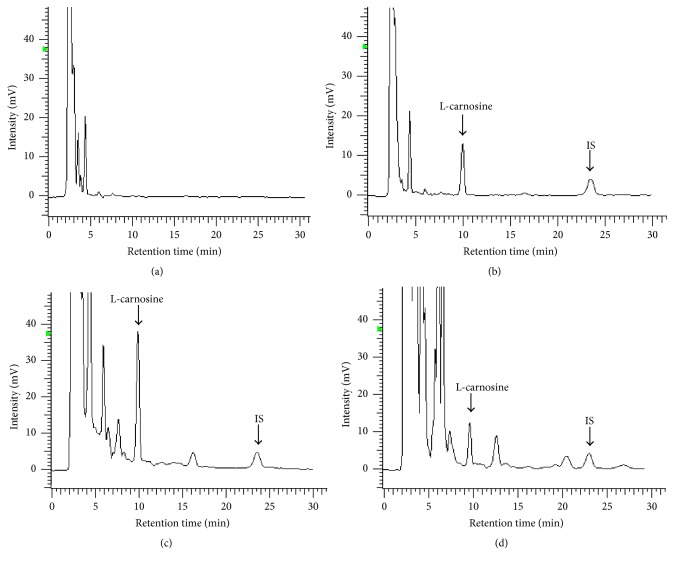
Representative chromatograms of diluted blank homogenates (a); diluted blank homogenates spiked with carnosine (10.16 *μ*g·mL^−1^) and IS (25 *μ*g·mL^−1^) (b); intact cardiac muscle homogenates of HCG rat with IS (c); intact cardiac muscle homogenates of DG rat with IS (d). Peak 1: L-carnosine. Peak 2: IS.

The carnosine and internal standard were completely separated under the chromatographic conditions employed. No endogenous interference was found at the retention times of carnosine and the IS in the diluted blank homogenates. Representative chromatograms for diluted blank homogenates, diluted blank homogenates spiked with carnosine (10.16 *μ*g·mL^−1^), IS (25 *μ*g·mL^−1^), intact cardiac muscle homogenates of HCG rat with IS, and intact cardiac muscle homogenates of DG rat with IS are shown in Figures 2(a)–2(d), respectively.

The standard calibration curves of carnosine were linear over the concentration range of 0.5–60.96 *μ*g·mL^−1^ for carnosine, with correlation coefficients above 0.99. The linear regression equation was *Y* = 0.10753*X* + 0.02851 (*r* = 0.9966), where *X* is the cardiac muscle concentration of carnosine and *Y* is the peak area ratio of carnosine to IS.

#### 3.4.2. Precision and Accuracy

As it was shown in Table 1, the intra- and interday precision presented (RSD%) were all less than 15.0%. The accuracy (RE%) of carnosine was less than 15%. These results indicated that the present method had good precision and accuracy.

#### 3.4.3. Recovery

The results of recoveries were shown in Table 2; recoveries ranged from 93.4 ± 1.5% to 98.8 ± 3.1%. The recovery of IS was 92.7 ± 3.2%.

#### 3.4.4. Stability

The stability of carnosines in rat cardiac muscle under different storage conditions is investigated which was shown in Table 3. The carnosines in rat cardiac muscle were stable at room temperature for 24 h and the concentration did not show any significant change after three freeze-thaw cycles and 1 month.

### 3.5. Application

**Figure 3:**
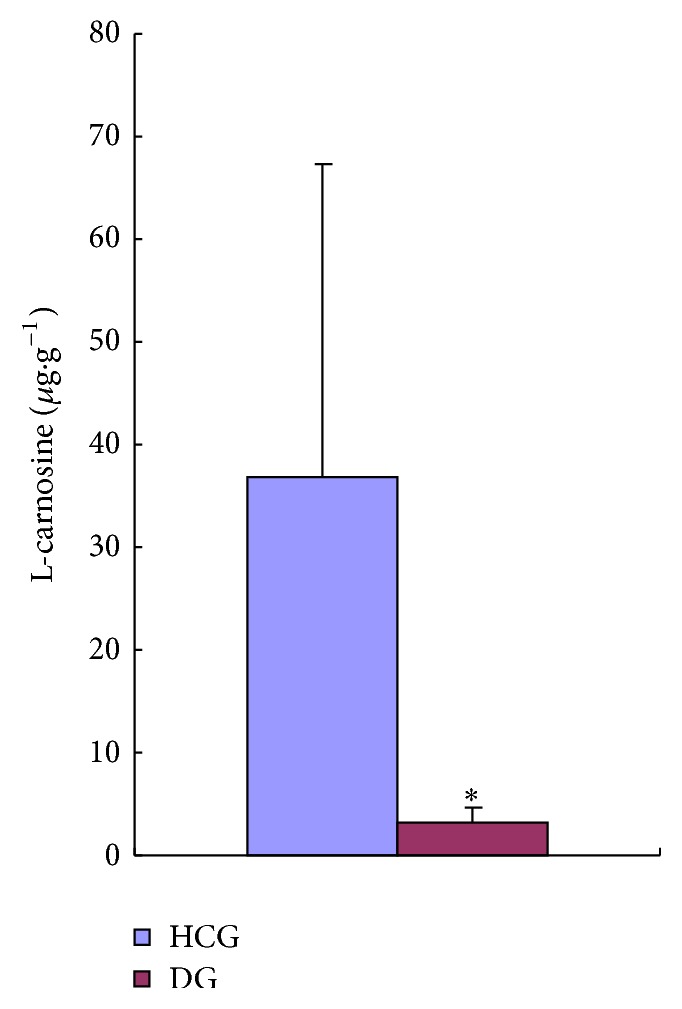
The concentration of L-carnosine in diabetes rat cardiac muscle (*n* = 5). Values are expressed as mean ± SD. ^*∗*^ *P* < 0.05 versus Healthy Control Group.

The validated method was applicated to assay of carnosine levels of DG and HCG rat cardiac muscle.  Table 4 and Figure 3 show the concentration of carnosine in the DG and HCG group. The level of carnosine was significantly lower in the DG group, compared to that in the HCG group (*P* < 0.05). Chromatogram of diabetes rat cardiac muscle shown in Figure 2 illustrates that peaks of endogenous substance increased obviously within retention time of 8 minutes.

Diabetes is closely related to various cardiovascular diseases which are the most frequent cause of death in diabetes patients. Recently, diabetic cardiomyopathy has received extensive attention. Although pathological mechanism is unclear, research has been concerned with the effect of myocardial cells abnormal metabolism among which oxidative stress is the main link of the pathogenesis leading to diabetes chronic complications [21–23]. The damage caused by diabetic oxidative stress showed that free radical is increasing and antioxygen is decreasing.

Previous reports have showed that L-carnosine is the important material involved in the maintenance of cardiac muscle intracellular physiological pH [24], as well as exciting contraction force of cardiac muscle cell [25]. L-carnosine is well known as an excellent antioxidant. According to our experimental results, the level of carnosine was low in the diabetes rats, and we propose antioxidant L-carnosine as a regulator involved in the metabolic response of myocardial cells in diabetic complications. Hipkiss and Chana [26] reported that L-carnosine protects the biological activity of some biological macromolecules by competitively reacting with aldehydes and ketones which are the substances to induce protein oxidation and cross-linked. Our results are consistent with the report above and confirm the truth furtherly. Therefore, L-carnosine can play an important role in prevention and improvement of the course of diabetic cardiomyopathy in the future.

We developed a simple and sensitive method to accurately quantify the level of endogenous carnosine in rat cardiac muscle. This method is an improvement on several existing methods of quantitation. Virtually most HPLC methods described in the literature use external standard method. A major advantage of our method is the use of the novel internal standard which ensured the analytical method accuracy. Moreover, equipment for HPLC-UV analysis is often a part of the existing standard instrumentation in most laboratories and staff is usually well experienced in its use. The UV detector is known for its stability and low demand in terms of maintenance. The method can be widely applicable for the determination of L-carnosine in biological matrices. Using the assay, we demonstrated that the content of carnosine was significantly lower in cardiac muscle of diabetes rats compared to the healthy control rats. The results showed that L-carnosine could be a regulator in diabetic complications.

## Figures and Tables

**Table 1:** Precision and accuracy of L-carnosine determination (intraday: *n* = 6; interday: *n* = 3 days with 6 replicates per day).

Added concentration	Intraday (*n* = 6)			Interday (*n* = 3 × 6)		
(*μ*g·mL^−1^)	Mean found (*μ*g·mL^−1^)	RE (%)	RSD (%)	Mean found (*μ*g·mL^−1^)	RE (%)	RSD (%)
1.02	0.96	−5.6	4.98	1.00	−1.8	8.3
8.13	8.56	5.29	1.74	8.04	−1.2	13.8
32.51	31.10	−4.3	4.71	32.15	−1.1	10.4

**Table 2:** Recoveries of L-carnosines and IS (*n* = 6).

Compound	Concentration (*μ*g·mL^−1^)	Recovery (%) (mean ± SD)	RSD (%)
L-carnosine	1.02	93.4 ± 1.5	1.6
	8.13	97.6 ± 3.7	3.8
	32.51	98.9 ± 3.1	3.1

IS	500	92.7 ± 3.2	3.5

**Table 3:** Stability of carnosine in rat cardiac muscle.

Concentration (*μ*g·mL^−1^)			Accuracy (%)	RSD (%)
	Added	Found (mean ± SD)		
Three freeze-thaw cycles	1.02	1.10 ± 0.08	7.7	7.03
	8.13	7.37 ± 0.22	−9.4	2.93
	32.51	36.51 ± 0.99	12.3	2.73

Room temperature for 24 h	1.02	0.97 ± 0.06	−4.9	5.74
	8.13	8.55 ± 0.73	5.2	8.56
	32.51	35.86 ± 2.05	10.3	5.71

1-month stability	1.02	1.04 ± 0.11	1.5	10.19
	8.13	8.42 ± 0.16	3.6	1.95
	32.51	35.16 ± 1.68	8.1	4.79

**Table 4:** Concentration of carnosine in rat cardiac muscle.

Healthy Control Group (*μ*g·g^−1^)	Diabetes Group (*μ*g·g^−1^)
79.8	1.54
57.7	1.77
10.5	3.27
23.1	3.03
13.6	5.67

## References

[1] Tian Y., Xie M., Wang W., Wu H., Fu Z., Lin L. (2007). Determination of carnosine in Black-Bone Silky Fowl (Gallus gallus domesticus Brisson) and common chicken by HPLC. *European Food Research and Technology*.

[2] Gil-Agustí M., Esteve-Romero J., Carda-Broch S. (2008). Anserine and carnosine determination in meat samples by pure micellar liquid chromatography. *Journal of Chromatography A*.

[3] Wideman J., Brink L., Stein S. (1978). New automated fluorometric peptide microassay for carnosine in mouse olfactory bulb. *Analytical Biochemistry*.

[4] Boldyrev A. A., Dupin A. M., Bunin A. Y., Babizhaev M. A., Severin S. E. (1987). The antioxidative properties of carnosine, a natural histidine containing dipeptide. *Biochemistry International*.

[5] Jay J. L., Miller D. J., Morrison J. D. (1990). Histidyl derivatives in rabbit lens and their diminution in human cataract. *Meetings Abstracts. Journal of Physiology (London)*.

[6] Boldyrev A., Abe H., Stvolinsky S., Tyulina O. (1995). Effects of carnosine and related compounds on generation of free oxygen species: a comparative study. *Comparative Biochemistry and Physiology Part B: Biochemistry and Molecular Biology*.

[7] Salim-Hanna M., Lissi E., Videla L. A. (1991). Free radical scavenging activity of carnosine. *Free Radical Research*.

[8] Boldyrev A. A., Dupin A. M., Pindel E. V., Severin S. E. (1988). Antioxidative properties of histidine-containing dipeptides from skeletal muscles of vertebrates. *Comparative Biochemistry and Physiology*.

[9] Decker E. A., Faraji H. (1990). Inhibition of lipid oxidation by carnosine. *Journal of the American Oil Chemists' Society*.

[10] Kohen R., Yamamoto Y., Cundy K. C., Ames B. N. (1988). Antioxidant activity of carnosine, homocarnosine, and anserine present in muscle and brain. *Proceedings of the National Academy of Sciences of the United States of America*.

[11] Decker E. A., Livisay S. A., Zhou S. (2000). A Re-evaluation of the antioxidant activity of purified carnosine. *Biochemistry*.

[12] Yun J., Parker C. J. (1965). The effect of carnosine on myofibrillar ATPase activity. *Biochimica et Biophysica Acta*.

[13] Bowen W. J. (1965). Effects of pH, buffers, carnosine, histidine, and *β*-alanine on the shortening of glycerol-treated muscle fibers. *Archives of Biochemistry and Biophysics*.

[14] Rusakov V. V., Dolgikh V. T. (1992). The effect of carnosin on rat hemodynamics and myocardium metabolism in the early postresuscitation period. *Bulletin of Experimental Biology and Medicine*.

[15] Lee J. W., Miyawaki H., Bobst E. V., Hester J. D., Ashraf M., Bobst A. M. (1999). Improved functional recovery of ischemic rat hearts due to singlet oxygen scavengers histidine and carnosine. *Journal of Molecular and Cellular Cardiology*.

[16] Nardiello D., Cataldi T. R. I. (2004). Determination of carnosine in feed and meat by high-performance anion-exchange chromatography with integrated pulsed amperometric detection. *Journal of Chromatography A*.

[17] Sri Kantha S., Takeuchi M., Watabe S., Ochi H. (2000). HPLC determination of carnosine in commercial canned soups and natural meat extracts. *LWT—Food Science and Technology*.

[18] Aldini G., Orioli M., Carini M., Facino R. M. (2004). Profiling histidine-containing dipeptides in rat tissues by liquid chromatography/electrospray ionization tandem mass spectrometry. *Journal of Mass Spectrometry*.

[19] Schönherr J. (2002). Analysis of products of animal origin in feeds by determination of carnosine and related dipeptides by high-performance liquid chromatography. *Journal of Agricultural and Food Chemistry*.

[20] Park Y. J., Volpe S. L., Decker E. A. (2005). Quantitation of carnosine in humans plasma after dietary consumption of beef. *Journal of Agricultural and Food Chemistry*.

[21] Filippo C. D., Cuzzocrea S., Rossi F., Marfella R., D'Amico M. (2006). Oxidative stress as the leading cause of acute myocardial infarction in diabetics. *Cardiovascular Drug Reviews*.

[22] Haidara M. A., Yassin H. Z., Rateb M., Ammar H., Zorkani M. A. (2006). Role of oxidative stress in development of cardiovascular complications in diabetes mellitus. *Current Vascular Pharmacology*.

[23] Mehta J. L., Rasouli N., Sinha A. K., Molavi B. (2006). Oxidative stress in diabetes: a mechanistic overview of its effects on atherogenesis and myocardial dysfunction. *International Journal of Biochemistry and Cell Biology*.

[24] Swietach P., Camelliti P., Hulikova A., Kohl P., Vaughan-Jones R. D. (2010). Spatial regulation of intracellular pH in multicellular strands of neonatal rat cardiomyocytes. *Cardiovascular Research*.

[25] Zaloga G. P., Roberts P. R., Black K. W. (1997). Carnosine is a novel peptide modulator of intracellular calcium and contractility in cardiac cells. *American Journal of Physiology—Heart and Circulatory Physiology*.

[26] Hipkiss A. R., Chana H. (1998). Carnosine protects proteins against methylglyoxal-mediated modifications. *Biochemical and Biophysical Research Communications*.

